# Identity-by-descent analysis of a large Tourette’s syndrome pedigree from Costa Rica implicates genes involved in neuronal development and signal transduction

**DOI:** 10.1038/s41380-022-01771-9

**Published:** 2022-10-12

**Authors:** Niamh Ryan, Cathal Ormond, Yi-Chieh Chang, Javier Contreras, Henriette Raventos, Michael Gill, Elizabeth Heron, Carol A. Mathews, Aiden Corvin

**Affiliations:** 1 Neuropsychiatric Genetics Research Group, Department of Psychiatry, Trinity College Dublin, Dublin, Ireland; 2 Department of Psychiatry, Center for OCD, Anxiety, and Related Disorders, University of Florida, Gainesville, FL USA; 3 Centro de Investigación en Biología Celular y Molecular, Universidad de Costa Rica, San José, Costa Rica; 4 School of Biology, Universidad de Costa Rica, San José, Costa Rica; 5 University of Florida Genetics Institute, University of Florida, Gainesville, FL USA

**Keywords:** Genetics, Psychiatric disorders

## Abstract

Tourette Syndrome (TS) is a heritable, early-onset neuropsychiatric disorder that typically begins in early childhood. Identifying rare genetic variants that make a significant contribution to risk in affected families may provide important insights into the molecular aetiology of this complex and heterogeneous syndrome. Here we present a whole-genome sequencing (WGS) analysis from the 11-generation pedigree (>500 individuals) of a densely affected Costa Rican family which shares ancestry from six founder pairs. By conducting an identity-by-descent (IBD) analysis using WGS data from 19 individuals from the extended pedigree we have identified putative risk haplotypes that were not seen in controls, and can be linked with four of the six founder pairs. Rare coding and non-coding variants present on the haplotypes and only seen in haplotype carriers show an enrichment in pathways such as *regulation of locomotion* and *signal transduction*, suggesting common mechanisms by which the haplotype-specific variants may be contributing to TS-risk in this pedigree. In particular we have identified a rare deleterious missense variation in *RAPGEF1* on a chromosome 9 haplotype and two ultra-rare deleterious intronic variants in *ERBB4* and *IKZF2* on the same chromosome 2 haplotype. All three genes play a role in neurodevelopment. This study, using WGS data in a pedigree-based approach, shows the importance of investigating both coding and non-coding variants to identify genes that may contribute to disease risk. Together, the genes and variants identified on the IBD haplotypes represent biologically relevant targets for investigation in other pedigree and population-based TS data.

## Introduction

Tourette syndrome (TS) is a substantially heritable (*r*^2^ = 0.6–0.8) [1] and phenotypically heterogeneous neuropsychiatric disorder that has a complex and multifactorial aetiology. The genetic architecture of TS is likely to involve a spectrum of risk variants from common single nucleotide polymorphisms (SNPs) [2, 3], to *de novo* coding variants [4] and rare copy number variants (CNVs) [5], implicating multiple genes (reviewed by [6]) and possible gene × environment interactions [7]. An investigation into the variance in liability to disease, based on genome-wide association study (GWAS) data [3], estimated that 21% of TS heritability was explained by variants with a minor allele frequency (MAF) between 0.001 and 0.05, suggesting that rare variants make an important contribution to risk ([8]; reviewed in [9]). Rare variant studies have suggested a role for genes involved in the histaminergic pathway [10–12] the cadherin signalling pathway [13, 14] and neurite outgrowth [15, 16]. However, the cohort sizes available are significantly smaller than for many other psychiatric disorders, which may explain why it has been challenging to conclusively replicate results. Understanding the molecular aetiology of TS may be important in developing better treatments and improving patient care [5]. TS lies on a spectrum of genetically related tic disorders [17], and more than 85% of TS patients have co-morbid neuropsychiatric diagnoses (e.g. attention deficit hyperactivity disorder (ADHD) or obsessive-compulsive disorder (OCD)) [18, 19], and this is likely to represent shared genetic liability [20], suggesting that improving our understanding of the genetics of TS may have wider implications.

Whole-genome sequencing (WGS) data from large, densely affected pedigrees can be used to study the full spectrum of genetic variation contributing to disease aetiology within a more homogenous genetic background. In particular, rare (MAF < 0.01) and ultra-rare (MAF < 0.001) pathogenic variants segregating with illness are likely to be enriched in families compared to population case-control cohorts [21, 22]. We report data from a large pedigree (>500 individuals) densely affected by TS and co-morbid psychiatric disorders from a genetically isolated Costa Rican population [23]. The pedigree spans eleven generations and shares ancestry from six founder pairs (FPs) (Suppl Fig. 1). All of the affected individuals within the pedigree are distantly related to each other (one pair separated by 7 meiotic steps, a second by 9 meiotic steps and the remaining separated by 12 or more meiotic steps), and descended from at least one (in some cases several) of the six FPs. Linkage analysis to look for variants co-segregating with illness in a sparse set of distantly related individuals is problematic due to the limitation posed by the lack of directly observed genotype data for individuals from generations further up the pedigree [24, 25]. Here we use identity-by-decent (IBD) analysis to investigate whether TS-affected individuals from this genetic isolate share identical segments of DNA (haplotypes) inherited from a common ancestor. The more distantly related a pair of individuals are (the more meiotic steps between individuals), the less IBD sharing is expected (exponentially fewer and shorter IBD segments). Three or more distantly related individuals sharing a haplotype IBD is unlikely and therefore noteworthy [24]. Similarly, if an IBD haplotype region is shared by affected individuals only and is not seen in pedigree or population controls, it is more likely that genetic variants on that IBD haplotype may be contributing to disease risk. The corollary of this is that if a haplotype is common, it may enter the pedigree from multiple independent events and consequently, is less likely to be associated with disease risk.

We have generated WGS data for 19 individuals from this extended pedigree (17 TS-affected and two controls) and performed an IBD analysis to identify regions of the genome inherited from common ancestors which may be contributing to TS in these individuals. We hypothesised that these IBD haplotype regions may contain rare and ultra-rare deleterious variants that are segregating with illness. By utilising whole-genome rather than whole exome sequencing technology, we are able to investigate the functional impact of non-coding as well as coding variants. This is an important advantage over studies that focus on coding regions [4, 13, 26] or candidate genes [27].

## Methods

The materials and methods are described in full in the Supplementary Information.

### Recruitment and diagnosis

#### TS pedigree

IRB approval was obtained for this study at all participating sites. Individuals with TS were recruited from health care professionals, media advertisements, assessments done in the schools, and family members who had heard of the study. TS probands and their parents were recruited into the study. TS probands underwent clinical assessment, and probands and their parents provided blood samples for genetic studies. Eighteen of the selected individuals had tic disorders (either TS or chronic motor/vocal tics (CMVT); eleven with a co-morbid ADHD diagnoses); one had OCD and one was identified as unaffected (Suppl Table 1). Affected status was defined as having either confirmed or probable TS or CMVT. Therefore 17 individuals were consider affected and two were considered controls (see Supplementary Methods: Diagnosis).

#### Controls

Genotype data for 91 Costa Rican control samples were acquired from two sources: 49 individuals from the Costa Rican Super Controls (genotyped using the Broad_GWAS_supplemental_15061359_A1 chip; courtesy of Henriette Raventos and Javier Contreras. *unpublished*); and 42 unaffected unrelated founder individuals from the Costa Rican bipolar disorder family study (genotyped on the Omni2.5M chip; https://www.nimhgenetics.org/download-tool/BP study 71; courtesy of Nelson Freimer) (see Supplementary Methods: Diagnosis).

### Pedigree whole-genome sequencing

DNA concentrations were quantified for twenty pedigree individuals by Qubit and the quality of DNA was determined by agarose gel electrophoresis. The DNA for individual 14 (diagnosis: TS, ADHD-probable) failed quality control metrics for sequencing and was excluded. WGS was performed by Edinburgh Genomics (Clinical Genomics) on a HiSeqX to an average depth of coverage of 30× per sample. All FASTQ files were examined using *FastQC* and *samtools* [28] to identify DNA contamination or degradation. Reads were aligned to the GRCh38 reference genome following the GATK Best Practices [29]. Briefly, this involved marking PCR duplicates, base quality score recalibration, local realignment of reads around indels, and variant calling with HaplotypeCaller. Genotype calling was performed jointly [30], and variant quality score recalibration (VQSR) was performed on the SNVs and Indels separately. Using the software *peddy* [31] all samples were jointly checked for: (i) relatedness discordance; (ii) sex discordance; (iii) low median coverage; and (iv) ancestry clustering by a principal component analysis (PCA) based on 1000 Genomes Project data (Supplementary Figs. 2 and 3) [32].

### IBD pipeline and filtering strategy

**Fig. 1:**
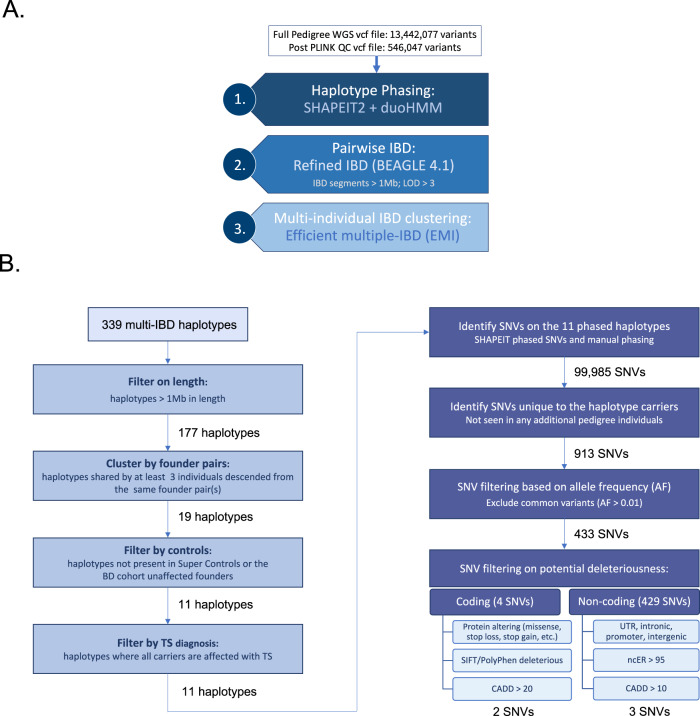
Outline of the analysis strategies implemented in this study. **A** IBD analysis pipeline: WGS data were pre-filtered to produce an LD-pruned set of common variants (MAF > 5%) for IBD analysis. Haplotype phasing was performed using SHAPEIT2 + duoHMM plug-in, pairwise IBD analysis was performed using the refined-IBD algorithm. Clusters of individuals sharing haplotypes IBD (multi-IBD clusters) were identified using the efficient multiple-IBD (EMI) algorithm. **B** Haplotype and variant filtering strategy: IBD haplotypes identified from the IBD pipeline were filtered on length, clustered by founder pair, filtered to exclude haplotypes seen in population controls and seen in unaffected pedigree individual. The most informative subset of haplotypes was taken forward to search for deleterious variants. Using this approach, the number of haplotypes to investigate was reduced from 339 to eleven, from which five deleterious haplotype-specific variants were identified (two coding and three non-coding).

To prepare the WGS data for the IBD analysis pipeline (Fig. 1A), the following QC filters were applied using PLINK [33]: genotype missing rate (<0.01); individual missing rate (<0.05); Hardy Weinberg equilibrium (<0.001); MAF filter (>0.05); LD-prune (window size: 500 kb; step size: 50; *r*^2^ threshold: 0.6); rsIDs only; and no Mendelian errors (for the single parent-offspring pair). This filtering reduced the number of single nucleotide variants (SNVs) included in the IBD analysis from 13,442,077 to 546,047. Haplotype phasing was performed using SHAPEIT2 + duoHMM plug-in [34], using the 1000 Genomes Phase 3 reference panel. Pairwise IBD analysis was performed using the refined-IBD algorithm; high confidence IBD segments were identified by filtering on IBD segment length (>1 Mb) and LOD scores, which are log base 10 of the likelihood ratio (default LOD > 3) [35]. An individual can carry 0, 1, or 2 copies of any specific haplotype (haplotype not present, present on one chromosome (IBD1), or present on both chromosomes (IBD2) respectively). The efficient multiple-IBD (EMI) algorithm [36] was used to identify IBD haplotypes that occur three or more times in the dataset (multi-IBD clusters, including individuals who are either IBD1 and IBD2 for the haplotype). The control samples were similarly processed using the PLINK QC filters described above (see Supplementary Methods) and investigated using our IBD analysis pipeline to find evidence of the putative disease-associated haplotypes identified in the TS pedigree.

**Fig. 2:**
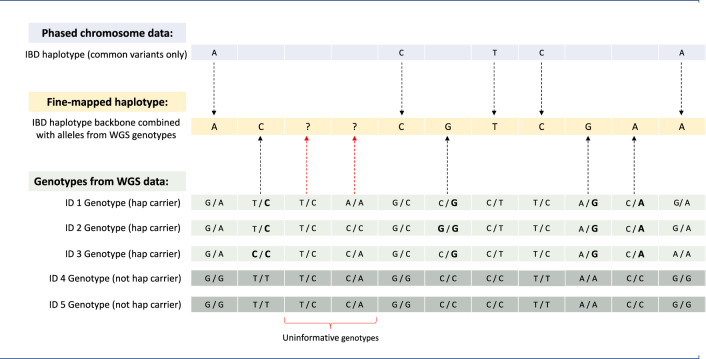
Fine-mapping of the IBD haplotypes (yellow block) using the phased chromosome data (blue; above the fine-mapped haplotype) and the WGS data (green/grey; below the fine-mapped haplotype). The arrows show the origin of the allele assigned to the fine-mapped haplotype. The backbone of the haplotype was built from the SNPs present on the IBD haplotype in the phased chromosome data. The remaining variants from the WGS data (~3.2% of the total variants on the haplotype) were mapped to the haplotype based on the genotype status of the IBD haplotype carriers (light green) versus the individuals who do not carry the IBD haplotype (grey), as demonstrated by the alleles highlighted in bold. Where a definitive haplotype allele could not be assigned due to uninformative genotypes, no allele was assigned at that position (WGS positions marked with red arrows).

The filtering pipeline outlined in Fig. 1B. was used to identify the most plausible putative risk haplotypes and any deleterious variants present on these risk haplotypes. Haplotypes were clustered by FP, to identify haplotypes shared by three or more individuals descended from the same founders; subset by length (>1 Mb); investigated using the two Costa Rican population control cohorts to identify haplotypes absent from both (Suppl Table 2); and filtered requiring a diagnosis of TS in all haplotype carriers. The boundaries of these haplotypes (originally identified using the LD-pruned data) were re-defined using the full phased chromosome data. Fine-mapping was performed using the WGS data to identify all SNVs present on the IBD haplotypes (Fig. 2; Supplementary Methods). Risk haplotype-specific variants (not seen in any additional pedigree individuals) were filtered on MAF (gnomAD.AMR.V3.1.1 [37]), excluding common variants (MAF > 0.01). Both coding and non-coding variants were filtered on CADD scores (PHRED-like scaled > 20 and >10 respectively, GRCh38-v1.6) [38]. Coding variants were further filtered on impact to protein structure (frameshift, nonsense, splice-site and missense variants) and deleteriousness as predicted by SIFT [39] and PolyPhen [40](taken from VEP [41]); while non-coding variants were filtered on ncER scores (non-coding essential regulation (V2) > 95 [42]). As ncER scores are only mapped to hg19, LiftOver was used to convert all of the rare non-coding variants from GRCh38 to hg19, excluding variants known to be unstable when converted [43]. Gene expression, network and gene-ontology analyses were performed using data from the Human Protein Atlas (http://www.proteinatlas.org; [44]); the STRING database of protein-protein interactions [45]; the Gene Ontology (GO) resource (http://geneontology.org/); and data from the PsychENCODE toolset (http://resource.psychencode.org/) (see Supplementary Methods).

## Results

The IBD analysis pipeline (Fig. 1A) identified 339 multi-IBD clusters (IBD haplotypes seen at least three times in the dataset). While the six FPs may be related to each other at some point in history (very likely considering the pedigree comes from an isolated population with a small number of known founders (~300 individuals)), it is not possible to know with any certainty which pairs might be related to each other, or the degree of relatedness. By extension, while some of the pedigree individuals are genetically more similar to each other than the expected relatedness from the pedigree (as cryptic relatedness is common in genetic isolates with a limited number of founder individuals; see Supplementary Information), the only confirmed relatedness between the affected individuals is through the pedigree and the known FPs. Therefore, each FP was treated as independent and this analysis focused on identifying regions of the genome shared IBD between descendants of the same FPs. Using the strategy outlined in Fig. 1B, the identified haplotypes were filtered, resulting in a list of eleven plausible putative risk haplotypes for further investigation (Table 1).

**Table 1:** Summary of the TS pedigree multi-IBD haplotypes: multi-IBD haplotype ID; genomic location (chromosome, start and end; GRCh38); length (Mb); identification numbers (ID)s of haplotype carriers; Founder Pairs (FPs) from which each haplotype carrier is descended; the primary (most common) FP(s) associated with the haplotype; number of individuals carrying the haplotype descended from the main FP(s); diagnoses of each haplotype carrier: TS (Tourette syndrome confirmed); OCD (obsessive-compulsive disorder confirmed); OCD prob (probable OCD diagnosis); ADHD (attention deficit hyperactivity disorder confirmed); ADHD prob (probable ADHD diagnosis); CMVT (chronic motor or verbal tic disorder).

Haplotype	Chr	Start	End	Length (Mb)	Haplotype carriers				Relatedness to founder pairs (FPs)				Main	# Carriers descended from main FP(s)	Diagnoses			
ID					ID1	ID2	ID3	ID4	ID1	ID2	ID3	ID4	FP(s)		ID1	ID2	ID3	ID4
1.1	1	44,527,042	48,054,945	3.53	12	6	19		B	A/B/C/D	A/B/C		B	3	TS/OCD prob/ADHD prob	TS	TS	–
2.1	2	13,000,487	15,987,485	2.99	12	6	9		B	A/B/C/D	B		B	3	TS/OCD prob/ADHD prob	TS	TS/ADHD prob	–
2.2	2	211,041,963	214,017,954	2.98	7	3	6	19	A/B	D	A/B/C/D	A/B	A/B	3	TS/ADHD	TS	TS	TS
4.1	4	154,028,117	164,629,683	10.6	2	1	6		D/F	C/D	A/B/C/D		D	3	TS	TS	TS	–
4.2	4	164,642,592	168,832,405	4.19	2	3	1		D/F	D	C/D		D	3	TS	TS	TS	–
5.1	5	107,466,596	110,400,753	2.93	10	7	12		B	A/B	B		B	3	TS/ADHD prob	TS/ADHD	TS/OCD prob/ADHD prob	–
6.1	6	11,239,710	15,065,694	3.83	1	15	19		C/D	C	A/B/C		C	3	TS	TS/ADHD	TS	–
9.1	9	101,864,325	106,732,943	4.87	7	7	15	6	A/B	A/B	C	A/B/C/D	A/B	3	TS/ADHD	TS/ADHD	TS/ADHD	TS
9.2	9	130,138,362	132,407,549	2.27	7	13	8		A/B	B	A/B/C		B	3	TS/ADHD	TS/ADHD	TS/ADHD	–
18.1	18	66,447,780	69,214,658	2.77	7	12	19		A/B	B	A/B/C		B	3	TS/ADHD	TS/OCD prob/ADHD prob	TS	–
20.1	20	9,153,822	12,651,396	3.5	5	1	6		C/E	C/D	A/B/C/D		C	3	CMVT/ADHD prob	TS	TS	–

Fine-mapping of the eleven putative risk haplotypes using the WGS data (Figs. 1B and 2) identified 433 rare (AF < 0.01) coding and non-coding variants specific to the haplotype carriers, of which 86 are ultra-rare (AF < 0.001) (Supplementary Table 3). Of these 433 rare variants, four are missense mutations; 254 are non-coding variants within the boundaries of 72 genes (intronic, UTRs and promoter regions); and 175 are intergenic. We filtered these variants using the predictors of deleteriousness: SIFT (deleterious); PolyPhen (damaging/probably damaging); CADD scores (coding and non-coding variants filtered on phred-scaled >20 and >10 respectively, representing the top 1% and 10% of predicted deleterious variants across the whole genome [46]); and ncER scores (non-coding essential regulation >95, ranking variants on predicted deleteriousness and representing the 95th percentile of putatively deleterious regulatory variants [42]). We identified five rare or ultra-rare putatively deleterious variants (Table 2), present on four haplotypes, shared by at least three affected individuals, altogether representing nine of the seventeen affected individuals from this pedigree, six of whom share ancestry with FP B (out of a maximum of nine individuals descended from FP B) (Table 3). For each of the five deleterious variants, the MAFs across all other populations in GnomAD were also investigated. For four of the five variants the MAF in AMR is the highest across any population, showing they are even rarer in other population groups. One variant, rs562279749, has a marginally higher MAF in the Finnish sample (FIN MAF = 0.001526), where it is rare rather than ultra-rare. In all other population groups it is either even rarer than in the AMR population or completely absent (Supplementary Table 4). This shows that all five deleterious variants have no substantial increase in frequencies across population cohorts.

**Table 2:** All putative deleterious variants that survived the variant filtering pipeline.

Haplotype ID	Position (GRCh38)	ID	REF	ALT	GnomAD AMR MAF	GnomAD minor allele	GnomAD MAX MAF	VEP consequence	VEP impact	GENE symbol	aa substitution	SIFT	PolyPhen	CADD Phred	pLoF score	PLoF missense Z-score	ncER Score
1.1	1_45617469	rs780636281	C	A	0.001392	A	0.00139153 (AMR)	Missense variant	Moderate	NASP	P/T	Deleterious (0.04)	Possibly damaging (0.621)	25.8	1	0.61	–
9.2	9_131619052	rs570357965	G	A	0.001976	A	0.00197628 (AMR)	Missense variant	Moderate	RAPGEF1	S/L	Deleterious low confidence (0)	Probably damaging (0.999)	23.8	1	3.13	–
2.2	2_211617876	rs1219527473	T	G	0.000661	G	0.000660599 (AMR)	Intron variant, downstream gene variant	Modifier	ERBB4	–	–	–	17.69	–	–	99.47
2.2	2_213098737	rs562279749	T	C	0.000661	C	0.00152643 (FIN)	Intron variant	Modifier	IKZF2	–	–	–	21.2	–	–	97.26
4.1	4_157889911	rs564274930	C	A	0.000513	A	0.00051267 (AMR)	Intron variant, non-coding transcript variant	Modifier	AC017037.5	–	–	–	16.67	–	–	95.03

Two rare amino acid substitutions (Latino/Admixed American (AMR) MAF < 0.01) and three ultra-rare intronic variants (AMR MAF < 0.001) were identified on four of the eleven putative risk haplotypes.

**Table 3:** All seventeen TS-affected individuals from the pedigree, showing from which founder pairs they are descended (one or several) and whether they carry the four haplotypes that contain the five deleterious variants.

Individual ID	Founder pairs	Hap 1.1	Hap 2.2	Hap 4.1	Hap 9.2
		(B)	(A/B)	(D)	(B)
		NASP	ERBB4 IKZF2	AC017037.5	RAPGEF1
1	C|D			X	
2	D|F			X	
3	D		X		
4	D				
5	C|E				
6	A|B|C|D	X	X	X	
7	A|B		X		X
8	A|B|C				X
9	A|B|C				
10	B				
11	B				
12	B	X			
13	B				X
15	C				
17	E				
19	A|B|C	X	X		
20	F				

Three of these individuals (IDs 6, 7 and 19) carry multiple haplotypes.

Two of the missense variations are predicted by SIFT and PolyPhen to be deleterious, with phred-scaled CADD scores greater than 20, suggesting they are in the top 1% of predicted deleterious variants across the genome [38]. rs570357965 (MAF: 0.001976) is located on chromosome 9 and results in a S/L amino acid substitution in the protein *RAPGEF1*. All three carriers share a diagnosis of TS and co-morbid ADHD and are 7th or more distant cousins (separated by at least 16 meiosis), sharing ancestry through FP B. *RAPGEF1* has a probability of being loss-of-function intolerant (pLI) score of 1 and an intolerance to missense variation Z-score of 3.13 [37, 47], implying that this gene is extremely intolerant to loss-of-function. rs780636281 (MAF: 0.001391) is located on chromosome 1 and results in a P/T substitution in the gene *NASP*. All three carriers share a diagnosis of TS, with one individual also diagnosed as ADHD-probable and OCD-probable. While the pedigree shows that these three individuals are at least 5th cousins, sharing ancestry through FP B, individuals 6 and 12 appear to be genetically more similar, with an observed relatedness closer to 2nd or 3rd cousins. Taken together with the fact that *NASP*, though also having a pLI score of 1, has a missense Z-score of 0.61 (suggesting it is more tolerant to missense variation than *RAPGEF1*) makes this a less interesting candidate for follow-on investigation.

Three non-coding variants had ncER scores greater than 95 and CADD scores greater than 10 (Table 2). All three variants are ultra-rare and intronic. rs1219527473 (MAF: 0.000661), located within intron 18 of *ERBB4* on chromosome 2 has a CADD score of 17 and an ncER score of 99.47, one of the highest confidence ncER percentiles. rs562279749 (MAF: 0.000661) is located within intron 4 of the gene *IKZF2*. This variant has a CADD score of 21.6, the highest CADD score for a rare non-coding variant on any of the risk haplotypes, ranking it as one of the top 1% potentially deleterious variants across the genome and comparable to the deleteriousness of the missense variants. Of note, both rs1219527473 and rs562279749 are located on the same chromosome 2 haplotype, carried by four individuals (two sharing ancestry with both FPs A and B; one descended from four FPs (A, B, C and D) and one descended solely from FP D). One of these individuals also carries the chromosome 1 haplotype 1.1 (*NASP*). It should be noted that while relatedness check confirmed 5 of the 6 pairwise relationships to be at least 5^th^ cousins, one pair (individuals 6 and 7) appear to be genetically more similar, with an observed relatedness closer to 2nd or 3rd cousins. Finally, rs564274930 (MAF: 0.000513) is located within intron 1 of the lncRNA gene *AC017037.5*, present on a chromosome 4 haplotype 4.1, carried by three individuals sharing ancestry with FP D. In addition to the chr4 haplotype, one of these individuals also carries both the chromosome 1 haplotype 1.1 (*NASP*) and the chromosome 2 haplotype 2.2 (*ERBB4/IKZF2*).

While the filtering pipeline focused our attention on the set of rare haplotype-specific variants with the strongest evidence for deleteriousness, these five variants only represent four of the eleven risk haplotypes, carried by nine of the affected individuals in the pedigree. We questioned whether the full set of rare variants, not just the most deleterious subset, across all eleven haplotypes may be connected through common networks and might implicate pathways that would not be seen when focusing only on the most stringent subset of haplotype genes. Therefore, we used protein-protein interaction (PPI) network analysis and gene-ontology (GO) enrichment analysis to investigate whether there were any functional links across the protein-coding genes with rare variations from the risk haplotypes. Specifically, we focused on the set of genes shown to be brain-expressed as being most functionally relevant.

Using data from the Human Protein Atlas, which incorporates expression data from three different resources (HPA, GTEx and FANTOM5) we determined that 66 of the 72 genes with rare and ultra-rare haplotype-specific variants are brain-expressed (Supplementary Data). Using STRING network analysis, 38 of these brain-expressed genes were found to be part of eight clusters containing two or more protein-coding genes (Suppl Fig. 4). The largest cluster consists of 12 genes, including *RAPGEF1* and *ERBB4*, connected by *ABL1* (Suppl Fig. 5). These connections are driven by a combination of known interactions (experimentally determined), predicted interactions, text-mining, protein homology and co-expression data. GO enrichment analysis of this set of 12 genes returned 102 FDR-significant GO terms [48], with the top three terms being *regulation of cell migration* (GO:0030334); *regulation of cell motility* (GO:2000145) and *regulation of locomotion* (GO:0040012) (Suppl Table 5). *IKZF2* clustered with four other proteins, while *NASP* clustered with *SMC2* (Suppl Fig. 5). However, GO analysis of these clusters did not return any FDR-significant terms, likely due to the limited number of genes included. Furthermore, these smaller clusters might be biased by the number of annotations available in the STRING database compared to the results of the full set of brain-expressed genes.

GO enrichment analysis of the full set of 66 brain-expressed genes from the eleven risk haplotypes returned 467 GO terms with uncorrected *p*-values <0.05, with top terms including: *response to nitrogen compound*; *regulation of MAPK cascade*; *transmembrane receptor protein tyrosine kinase signalling pathway; cellular protein modification process; positive regulation of protein phosphorylation*; *macromolecule modification*; *regulation of locomotion*; *positive regulation of kinase activity*; *tube development; and nerve growth factor signalling pathway* (Supplementary information; Supplementary Table 6). Of these genes, 51 are in psychiatric disorder-associated gene co-expression modules from PsychENCODE (http://resource.psychencode.org/; [49]) (Supplementary information; Supplementary Table 7).

## Discussion

We report a WGS study from a large TS pedigree (>500 individuals) from a Costa Rican population isolate. We hypothesized that there would be an enrichment of rare variants on founder haplotypes unique to the pedigree (not seen in population controls), shared by subsets of the affected individuals descended from the same founders. As rare variants are normally thought to have a higher impact on disease risk, we have focused our investigation on the set of variants with the highest predicted deleteriousness scores (CADD score, impact on coding sequence, etc). We theorised that the genes carrying these rare, deleterious, haplotype-specific variants might be functionally connected, giving an insight into the aetiology of TS in this pedigree. By using WGS we were able to investigate both coding and non-coding haplotype-specific variants in this pedigree, rather than the exclusive analysis of coding variants in more typical whole exome studies [13]. This is important, as other studies have implicated regulatory variants in neuropsychiatric disorders, including TS [2, 3], although the challenges of interpreting such data are well described [50]. Recently, several well-characterised and stringently tested tools have been designed to predict deleteriousness of non-coding variation. Such metrics of deleteriousness, similar to those designed for coding variants, can help add confidence and clarity to the likelihood of a non-coding variant having a functional and putatively deleterious effect. In this study we used two methods, CADD and ncER, to identify the subset of non-coding variants most likely to be contributing to TS in this pedigree. To the best of our knowledge, this is the first time that these two methods have been combined in this way, adding an extra stringency to the filtering of non-coding variation.

The amount of expected IBD sharing and the corresponding length of chromosomal segments shared IBD between two related individuals can be estimated [24]. However, individuals with ancestry derived from genetically isolated populations such as the Central Valley of Costa Rica are likely to have a greater amount of IBD sharing than individuals from an outbred population. Calculating the expected amount of IBD sharing across multiple distantly related individuals becomes progressively more complicated, but equally the probability of sharing becomes more unlikely. Nevertheless, multiple individuals separated by 12 or more meiotic steps sharing the same region IBD is increasingly improbable and therefore more noteworthy. We identified eleven IBD haplotypes, each carried by at least three individuals with TS, all distantly related to each other (separated by at least 12 meiotic steps) through four of the six FPs, altogether representing fourteen of the seventeen affected individuals. By fine-mapping these unique haplotypes, we identified rare variants specific to the haplotype carriers. These variants were filtered on putative deleteriousness to identify the subset most likely to be contributing to TS aetiology (Fig. 1). This identified two rare missense variations (in *RAPGEF1* and *NASP*) and three ultra-rare intronic variants (in *ERBB4*, *IKZF2* and *AC017037.5*), only seen in the haplotype carriers. The haplotypes carrying these variants represent four of the eleven putative risk haplotypes. Nine of the seventeen affected individuals carry at least one of these haplotypes, with six of these individuals sharing ancestry with FP B. While there is no evidence supporting the involvement of either *NASP* or *AC017037.5* in neuropsychiatric disorders, *RAPGEF1*, *ERBB4* and *IKZF2* represent biologically relevant candidates.

*RAPGEF1*, also known as *C3G*, is a brain-expressed gene that is responsible for Rap1 activation downstream of the Reelin signalling and plays a crucial role in neural development, in particular radial glial attachment and neuronal migration [51, 52] . Dosage alterations in *RAPGEF1* have been associated with cerebral palsy [53] and a missense variant (c.423G>A (NM_198679.1, NP_941372.1:p.[M141I])) was found to be associated with neuropsychiatric symptoms in two siblings from a Pakistani pedigree (moderate intellectual disability, mood swings, repetitive behaviour and speech issues in one individual). A *rapgef1* zebrafish model identified a role in both brain and blood vessel development and showed that knockdown of Rapgef1 negatively influences locomotor capacity and motor neuron axon function [54]. Mouse models have shown that mice deficient in RAPGEF1 have an increased level of nuclear beta-catenin and increased neuronal precursor cell proliferation in the cerebral cortex [55] with impaired cortical neuron migration [51]. *ERBB4* is also brain-expressed and is a receptor for Neuregulin-1 (NRG1). Together the NRG1-ErbB4 pathway has been shown to be crucial for brain development, regulating the assembly of neural circuitry, myelination, neurotransmission, and synaptic plasticity [56]. Increased NRG1-ErbB4 protein levels in temporal cortex have been seen in patients with symptomatic epilepsy [57]. Furthermore, ErbB4 signalling has been shown to regulate top-down attention in mice, suggesting it may play a role in ADHD [58]. ErbB4 has also been shown in mouse models to play a role in long-term plasticity at inhibitory synapses from PV expressing interneurons, and that this plasticity is involved in the emergence of social memory during late adolescence [59]. The NRG1-ErbB4 pathway has been implicated in early schizophrenia association studies [60–63] and rare structural variants in the *ERBB4* gene have been identified in ASD [64], however, these results have not been replicated. IKZF2, also known as Helios, is a transcription factor (Zinc-finger) that has recently been shown to play a role in neuronal development, specifically, in the maturation of CA1 neuronal sub-cell population [65]. Helios null mice have also been shown to display schizophrenia-like symptoms.

Family-based WGS projects play an important role in helping us elucidate the complex genetic architecture of psychiatric disorders, allowing the full spectrum of genetic variations to be investigated simultaneously. Furthermore, rare, high-effect variations associated with illness can be found to be enriched in large pedigrees, increasing statistical power to identify such variants compared to population studies. We have identified five deleterious variants across four haplotypes that suggest potential mechanisms by which rare and ultra-rare variants on IBD haplotypes may potentially contribute towards TS aetiology in this extended pedigree. We have compiled converging evidence supporting the interaction of genes through common networks; the role of several of these genes in other neuropsychiatric disorders; expression of these genes in relevant tissues and cell types (excitatory and inhibitory neurons); and enrichment in relevant GO terms. Of particular note, *RAPGEF1*, *ERBB4* and *IKZF2* are all supported by independent studies to potentially play a role in neuropsychiatric phenotypes and are functionally connected to other genes with rare haplotype-specific variants (Suppl Fig. 6). These findings, though tentative, point to an intriguing pattern of functional connection between the genes with rare and ultra-rare haplotype-specific variants present in our analysis.

## Supplementary information

Supplementary Information

Supplementary Data

## Data Availability

The WGS data generated for the TS pedigree have been uploaded to the NIHM Data Archive (NDA, https://nda.nih.gov/) with the accession number 10.15154/1527895 (10.15154/1527895). The genotype data for the BD controls are available from the NIHM Repository & Genomics resource, Bipolar Disorder 12.3 (https://www.nimhgenetics.org/download-tool/BP), as part of Study 71 (Bipolar Endophenotypes in Population Isolates). The pipeline used to generate and investigate IBD haplotypes is available at https://github.com/R-niamh/IBD_analysis.
