# Supplementary material for: Identity-by-descent analysis of a large Tourette’s syndrome pedigree from Costa Rica implicates genes involved in neuronal development and signal transduction
Source: Mol Psychiatry. 2022 Oct 12;27(12):5020–7. doi: 10.1038/s41380-022-01771-9 (PMC9763103; doi:10.1038/s41380-022-01771-9)
Supplement: Supplementary file 1 — Supplementary Information [file 41380_2022_1771_MOESM1_ESM.doc]

**Supplementary Information**

Identity-by-descent analysis of a large Tourette’s syndrome pedigree from Costa Rica implicates genes involved in neuronal development and signal transduction. Ryan et al.

Table of Contents

[***Supplementary Methods: 1***](#__RefHeading___Toc79844935)

[Diagnosis: 1](#__RefHeading___Toc79844936)

[Ancestry determination/genealogical reconstruction: 1](#__RefHeading___Toc79844937)

[IBD analysis of the Costa Rican control datasets: 1](#__RefHeading___Toc79844938)

[Fine-mapping haplotypes and haplotype boundaries: 2](#__RefHeading___Toc79844939)

[Gene expression, network and gene ontology analysis: 2](#__RefHeading___Toc79844940)

[Supplementary Information: 3](#__RefHeading___Toc79844941)

[Peddy (PCA and relatedness): 3](#__RefHeading___Toc79844942)

*Fine-mapping of the putative TS risk haplotypes*[: 3](#__RefHeading___Toc79844943)

[Network and gene ontology enrichment analysis: 4](#__RefHeading___Toc79844944)

[Supplementary References: 6](#__RefHeading___Toc79844945)

[Supplementary Figures and Tables: 8](#__RefHeading___Toc79844946)

# Supplementary Methods:

## Diagnosis:

*TS pedigree*: Clinical assessments were conducted by a psychiatrist using semi-structured standardized instruments to assess for tic disorders (including Tourette syndrome (TS) and chronic motor/vocal tics (CMVT)), obsessive compulsive disorder (OCD), and attention deficit hyperactivity disorder (ADHD). Medical records were obtained for additional clinical information, when available. Best estimate diagnoses were made by two independent clinicians using all available clinical data, and final consensus diagnoses were made according to DSM-IV-TR criteria(1). Probable diagnoses were assigned if all symptom criteria for the disorder were met, but the required impairment or distress criterion could not be definitively determined. While individual 16 comes from a separate branch of the extended TS pedigree and is not directly related to any of the 6 founder pairs in this study, they are still ancestrally related to the affected pedigree individuals (as shown by the PCA analysis where ID16 clustered with the other pedigree individuals). Therefore, this individual was included in the IBD analysis, along with the unaffected pedigree individual 18, to improve the accuracy of haplotype phasing (by increasing sample size) and to aid the exclusion of pedigree-specific haplotypes unrelated to TS phenotype. Furthermore, as individual 18 is a parent of individual 5 (diagnosis: CMVT), their inclusion in the study allowed for the exclusion of mendelian errors.

*Controls*: Costa Rican super controls screened negative for the presence of psychiatric illnesses, including tic disorders, OCD, and ADHD. Unaffected unrelated family members from the BD study were screened for the absence of psychiatric disorders (not including tic disorders).

## Ancestry determination/genealogical reconstruction:

Ancestry of each TS proband was determined through the great-grandparents’ generation. TS probands were included in the study if they had five of eight grandparents who were born in the Central Valley of Costa Rica (CVCR), indicating likely descent from a small number of founders originating in the 1500s(2). Confirmation of ancestry was conducted via inspection of publicly available birth and death records housed either in the Costa Rican National Registry, or in local church records, with permission of the participants and their families. Reconstruction of the extended pedigree connecting family trios with previously unknown relationships was subsequently conducted using the information obtained from these historical records(2). The same ancestry requirements and confirmation processes were in place for the Costa Rican super control participants as for the TS pedigree individuals.

Ancestry of the TS pedigree individuals was confirmed using the software tool *peddy*, as described in the main Methods. Principal component analysis (PCA) using PLINK (v1.9) was performed to confirm the ancestry of the Costa Rican control cohorts compared against the 2,504 individuals from the 1000 Genomes Project Phase 3 dataset (1KG_Phase3_ALL.GRCh38.genotypes.20170504.vcf.gz and *20130606_g1k.ped*). The 1000 genomes data was QC’d to: remove multi-allelic (strand ambiguous) sites; variants with missing greater than 0.01; individuals with missing genotypes greater than 0.05; and variants with a minor allele frequency less than 0.01. Remaining variants were LD pruned (--indep 50 5 1.5). As the two cohorts were genotyped with different and largely non-overlapping genotype arrays, two separate PCA analyses were performed, on the subset of variants overlapping between the two control datasets and the cleaned 1000 genomes variants (Super controls vs 1000 Genomes: 93,701 variants; BD controls vs 1000 Genomes: 760,210 variants). For comparison against the control PCA analyses, the same pipeline was used to run a PCA of the pedigree against the 1000 Genomes dataset using plink (TS pedigree vs 1000 Genomes: 4,592,924 variants).

## IBD analysis of the Costa Rican control datasets:

The control samples were processed using the following PLINK QC filters: autosomal variants only; genotype missing rate (<0.01); individual missing rate (<0.05); Hardy Weinberg equilibrium (<0.001); and minor allele frequency (>0.05); LD pruning (window size: 500kb; step size: 50; r^2^ threshold: 0.6). rsIDs were used to convert variants from hg19 to hg38 using the 1KG Phase3 hg38 EUR dataset (only variants with rs numbers in both genome build datasets were converted). We ran the filtered and LD pruned dataset through our IBD analysis pipeline (SHAPEIT, refined-IBD, EMI) and looked for evidence of the putative disease-associated haplotypes identified in the TS pedigree.

## Fine-mapping haplotypes and haplotype boundaries:

The multi-IBD clusters were manually investigated in the phased chromosome files to more accurately define the boundaries of the IBD haplotypes. Where a break point predicted by refined IBD appeared to be caused by a single variation on a single chromosome, the variation was ignored and the break-point was reassigned at the point where the chromosomes had consistent and extensive difference in haplotype alleles (two or more alleles differing from the main haplotype), suggesting that a recombination event had occurred and the chromosome segment had moved on to an adjoining haplotype. The full WGS pedigree dataset was filtered on: autosomal SNVs only; genotyping rates (genotype missing rate < 0.01; individual missing rate < 0.05); HWE (HWE < 0.001) and MAF (excluding variants only seen on two chromosomes). Thus, all variants mapped to the disease haplotypes (the variants phased using the 1000 Genomes reference panel and the manually phased variants) were QC filtered using the same parameters. Mendelian errors related to the single parent-offspring pair (ID18 and ID5) were filtered out using the plink --mendel-duos command. No incongruencies were seen between the WGS genotypes and phased IBD haplotypes.

The phased variants were used to annotate the majority of variants on the IBD haplotypes (upper portion of main text Figure 2). Alleles for the unphased variants (which consisted of variants absent from the 1000 Genomes Phase 3 reference panel used to phase the chromosomes), representing on average 3.2% of the WGS data, were mapped to the IBD haplotypes based on the genotypes of the IBD haplotype carriers. Alleles unique to the haplotype carriers not seen in the other family members were taken as being present on the IBD haplotype (light green genotypes in Figure 2.). For genotypes where the haplotype carriers did not share the same allele (some homozygous REF, others homozygous ALT) no allele could be mapped to the haplotype (labelled as uninformative genotypes in Figure 2).

## Gene expression, network and gene ontology analysis:

Transcriptomics data was downloaded from the Human Protein Atlas ([*http://www.proteinatlas.org*](http://www.proteinatlas.org/);(3)), which combines gene expression data from three different resources: HPA(3), GTEx(4) and FANTOM5(5). Consensus transcript expression levels were summarized per gene in 62 tissues, including ten brain tissues, based on transcriptomics data from these three sources. The consensus normalized expression (“NX”) value is calculated as the maximum NX value for each gene in the three data sources. Genes were annotated as being brain expressed if they had NX>1 in at least one of the ten brain tissues.

The STRING database of protein-protein interactions allows for both physical and functional interactions between proteins to be assessed(6). Using the online browser (STRING v11, default parameters), all of the brain-expressed protein-coding genes containing rare and ultra-rare haplotype variants were assessed for direct (physical) and indirect (functional) interactions.

The Gene Ontology (GO) resource ([*http://geneontology.org/*](http://geneontology.org/)) is the largest repository of functional information on genes and gene products in the world(7, 8). In particular, this resource provides tools to investigate enrichment of GO terms in a set of genes and gene products compared to a background set of genes (9). Using the PANTHER Overrepresentation Test (Released 20210224) the protein-coding genes harbouring rare and ultra-rare variants were investigated for enrichment of GO terms compared to a reference list of genes (the rest of the genome). In addition, the subset of genes encoding proteins that were shown by STRING to be part of a network (original set, excluding singleton protein-coding genes) were investigated for GO enrichment.

The PsychENCODE Consortium was established to improve our understanding of the action of regulatory genomic elements in individuals with neuropsychiatric disorders. The data resources generated as part of this project are publicly available and have been promoted as providing insights into the biology of the developing, adult, and diseased human brain ([http://psychencodestg](http://psychencodestg/).wpengine.com/). We investigated whether the genes identified in this analysis were part of gene co-expression modules enriched across one or several psychiatric disorders as part of the PGC cross-disorder study(10). These modules of genes have been shown to be associated with schizophrenia (SCZ), bipolar disorder (BD) and autism spectrum disorder (ASD) and were calculated using Weighted Gene Co-Expression Analysis (WGCNA) on PsychENCODE RNA-seq samples(11) and made available as part of the PsychENCODE toolset ([*http://resource.psychencode.org/*](http://resource.psychencode.org/))*.*

# Supplementary Information:

## PCA and relatedness:

The genetic background of the TS pedigree individuals and the two Costa Rican control populations were assessed using PCA analysis against the 1000 Genomes Project data using plink (12). PCA analysis showed that, along with the TS pedigree individuals, the individuals from both control cohorts cluster with the Latino/Admixed American (AMR) population of the 1000 Genomes Project (Supplementary Figure 2), therefore confirming the usefulness of these controls for the IBD analysis.

Using the software *peddy(13)* all pedigree samples were jointly checked for: i) relatedness discordance; ii) sex discordance; and iii) low median coverage. Relatedness levels as calculated by *peddy(13)* are presented in Supplementary *Figure 4 and tabulated in the Supplementary Data.* A parent offspring pair would have an expected relatedness coefficient (*r*) of 0.5, while 4^th^ cousins (separated by 10 meiosis) would have an expected *r* of about 0.00195, 5^th^ cousins (separated by 12 meiosis) would have an expected R of ~0.000488, and 9^th^ cousins (20 meiosis) would have an expected *r* close to zero. The parent-offspring pair had an observed relatedness coefficient (r) of 0.5. Of the remaining 170 pairwise combinations, 99 had an observed relatedness the same or less than the expected relatedness (*r* between -0.09243 and 0) and 71 had a greater than expected relatedness (*r* between 0.001616 and 0.054), reflecting the homogenous genetic background of this isolated population and the likelihood that the married-in individuals were also related to the pedigree through relationships and ancestors not annotated in the pedigree.

## *Fine-mapping of the putative TS-risk haplotypes*:

The homolog of *RAPGEF1*, *RAPGEF2*, is a specific activator of D1 Dopamine Receptor-Dependent ERK Phosphorylation in mouse brain(14) and is crucial for the development of neural progenitor cells(15). *RAPGEF2* is also extremely intolerant to loss of function mutations and has been reported as being potentially causal in a pedigree affected by familial myoclonic epilepsy (OMIM#609530;(16)). We identified two rare non-coding *RAPGEF2* variants specific to haplotype carriers in this pedigree (Supplementary Data), however neither variant survived our strict filters of deleteriousness.

The rare missense variant rs780636281 in *NASP*, identified on haplotype 1.1, is carried by three individuals sharing ancestry with founder pair B. This gene encodes a histone chaperone protein which coordinates the assembly of stable H3-H4 dimers(17). While this gene has not been shown to have any role in neurodevelopmental or neuropsychiatric disorders, there is emerging evidence that histone turnover and chromatin assembly play an important role in neurological development and disease ((18);(19)) and the histone variant H3.3 may play be important for brain plasticity and neuronal function (20).

rs564274930, located in the 1^st^ intron of *AC017037*.*5*, is present on the chromosome 4 haplotype 4.1 carried by three individuals, all sharing ancestry with founder pair D. Little is known about the function or expression of the lincRNA *AC017037.5*, which was not found in the database of lncRNA expression LncExpDB ([*https://bigd.big.ac.cn/lncexpdb*](https://bigd.big.ac.cn/lncexpdb))(21).

## Network and gene ontology enrichment analysis:

Next we performed a gene set analysis of all 66 brain-expressed genes carrying haplotype-specific rare variants from all eleven putative risk haplotypes. Using STRING analysis (V11; (6)), *RAPGEF1* and *ERBB4* were shown to be connected by *ABL1*, another gene with a rare intronic variant specific to the haplotype carriers in this study (see Supplementary Figure 6 for the full output from the STRING Network analysis). Along with nine other genes in this set, these three genes form a network of 12 genes functionally linked by 102 FDR-significant gene ontology terms(9), including *regulation of cell migration* (GO:0030334); *regulation of cell motility* (GO:2000145) and *regulation of locomotion* (GO:0040012).

From the STRING analysis, *IKZF2* was also shown to be part of a network of five proteins from the haplotype analysis, along with *FSTL5*, *GRIA2*, *SPOCK3* and *ANXA10*. *FSTL5* has been found to be a genome-wide significant hit for bipolar disorder(22) and is important in cell development, synaptic transmission and plasticity((23);(24)) . *GRIA2* encodes a subunit of the AMPA sensitive glutamate receptor (GluA2) that functions as ligand-gated ion channel in the central nervous system and plays an important role in excitatory synaptic transmission. *GRIA2* has been implicated in a number of neuropsychiatric phenotypes including schizophrenia(25).

Both rs1219527473 and rs1219527473 (the *ERBB4* and *IKZF2* variants) are present on the same ~3Mb haplotype on chromosome 2, carried by four individuals from this pedigree. While there was no evidence from the literature or STRING analysis that *ERBB4* and *IKZF2* are functionally linked, it is worth questioning whether a transcription factor known to function in neuronal development and a gene also known to be functionally important in neuronal development could have an as yet unknown functional connection.

GO enrichment analysis of the full set of 66 genes with rare haplotype specific variants returned 416 GO terms with uncorrected p-values <0.05 (Supplementary Data; Supplementary Table 5). Furthermore, of the 66 genes investigated, 51 are in psychiatric disorder associated gene co-expression modules from PsychENCODE ([*http://resource.psychencode.org/*](http://resource.psychencode.org/); (11)). Seven of these genes (Supplementary Table 6) are in modules with GO terms related to neuronal function (including trans-synaptic signaling, synapse, myelin sheath, neuron projection development, axon guidance, etc.). The modules containing these seven genes are associated with ASD, BD, SCZ or a combination of these disorders. Five of the seven genes are part of modules that have enriched expression in excitatory and inhibitory neurons and the other two are part of modules enriched in GWAS hits.

# Supplementary References:

1. Mathews CA, Jang KL, Herrera LD, Lowe TL, Budman CL, Erenberg G, et al. Tic symptom profiles in subjects with Tourette Syndrome from two genetically isolated populations. Biol Psychiatry. 2007;61(3):292-300.

2. Mathews CA, Reus VI, Bejarano J, Escamilla MA, Fournier E, Herrera LD, et al. Genetic studies of neuropsychiatric disorders in Costa Rica: a model for the use of isolated populations. Psychiatr Genet. 2004;14(1):13-23.

3. Uhlen M, Fagerberg L, Hallstrom BM, Lindskog C, Oksvold P, Mardinoglu A, et al. Proteomics. Tissue-based map of the human proteome. Science. 2015;347(6220):1260419.

4. Consortium GT. The Genotype-Tissue Expression (GTEx) project. Nat Genet. 2013;45(6):580-5.

5. Lizio M, Harshbarger J, Shimoji H, Severin J, Kasukawa T, Sahin S, et al. Gateways to the FANTOM5 promoter level mammalian expression atlas. Genome Biol. 2015;16:22.

6. Szklarczyk D, Gable AL, Lyon D, Junge A, Wyder S, Huerta-Cepas J, et al. STRING v11: protein-protein association networks with increased coverage, supporting functional discovery in genome-wide experimental datasets. Nucleic Acids Res. 2019;47(D1):D607-D13.

7. Ashburner M, Ball CA, Blake JA, Botstein D, Butler H, Cherry JM, et al. Gene ontology: tool for the unification of biology. The Gene Ontology Consortium. Nat Genet. 2000;25(1):25-9.

8. Gene Ontology C. The Gene Ontology resource: enriching a GOld mine. Nucleic Acids Res. 2021;49(D1):D325-D34.

9. Mi H, Muruganujan A, Ebert D, Huang X, Thomas PD. PANTHER version 14: more genomes, a new PANTHER GO-slim and improvements in enrichment analysis tools. Nucleic Acids Res. 2019;47(D1):D419-D26.

10. Gandal MJ, Haney JR, Parikshak NN, Leppa V, Ramaswami G, Hartl C, et al. Shared molecular neuropathology across major psychiatric disorders parallels polygenic overlap. Science. 2018;359(6376):693-7.

11. Gandal MJ, Zhang P, Hadjimichael E, Walker RL, Chen C, Liu S, et al. Transcriptome-wide isoform-level dysregulation in ASD, schizophrenia, and bipolar disorder. Science. 2018;362(6420).

12. Chang CC, Chow CC, Tellier LC, Vattikuti S, Purcell SM, Lee JJ. Second-generation PLINK: rising to the challenge of larger and richer datasets. Gigascience. 2015;4:7.

13. Pedersen BS, Quinlan AR. Who's Who? Detecting and Resolving Sample Anomalies in Human DNA Sequencing Studies with Peddy. Am J Hum Genet. 2017;100(3):406-13.

14. Jiang SZ, Xu W, Emery AC, Gerfen CR, Eiden MV, Eiden LE. NCS-Rapgef2, the Protein Product of the Neuronal Rapgef2 Gene, Is a Specific Activator of D1 Dopamine Receptor-Dependent ERK Phosphorylation in Mouse Brain. eNeuro. 2017;4(5).

15. Maeta K, Edamatsu H, Nishihara K, Ikutomo J, Bilasy SE, Kataoka T. Crucial Role of Rapgef2 and Rapgef6, a Family of Guanine Nucleotide Exchange Factors for Rap1 Small GTPase, in Formation of Apical Surface Adherens Junctions and Neural Progenitor Development in the Mouse Cerebral Cortex. eNeuro. 2016;3(3).

16. Ohtsuka T, Hata Y, Ide N, Yasuda T, Inoue E, Inoue T, et al. nRap GEP: a novel neural GDP/GTP exchange protein for rap1 small G protein that interacts with synaptic scaffolding molecule (S-SCAM). Biochem Biophys Res Commun. 1999;265(1):38-44.

17. Cook AJ, Gurard-Levin ZA, Vassias I, Almouzni G. A specific function for the histone chaperone NASP to fine-tune a reservoir of soluble H3-H4 in the histone supply chain. Mol Cell. 2011;44(6):918-27.

18. Mossink B, Negwer M, Schubert D, Nadif Kasri N. The emerging role of chromatin remodelers in neurodevelopmental disorders: a developmental perspective. Cell Mol Life Sci. 2021;78(6):2517-63.

19. Wenderski W, Maze I. Histone turnover and chromatin accessibility: Critical mediators of neurological development, plasticity, and disease. Bioessays. 2016;38(5):410-9.

20. Bano D, Piazzesi A, Salomoni P, Nicotera P. The histone variant H3.3 claims its place in the crowded scene of epigenetics. Aging (Albany NY). 2017;9(3):602-14.

21. Li Z, Liu L, Jiang S, Li Q, Feng C, Du Q, et al. LncExpDB: an expression database of human long non-coding RNAs. Nucleic Acids Res. 2021;49(D1):D962-D8.

22. Stahl EA, Breen G, Forstner AJ, McQuillin A, Ripke S, Trubetskoy V, et al. Genome-wide association study identifies 30 loci associated with bipolar disorder. Nat Genet. 2019;51(5):793-803.

23. Maguschak KA, Ressler KJ. A role for WNT/beta-catenin signaling in the neural mechanisms of behavior. J Neuroimmune Pharmacol. 2012;7(4):763-73.

24. Zhang D, Ma X, Sun W, Cui P, Lu Z. Down-regulated FSTL5 promotes cell proliferation and survival by affecting Wnt/beta-catenin signaling in hepatocellular carcinoma. Int J Clin Exp Pathol. 2015;8(3):3386-94.

25. Alkelai A, Shohat S, Greenbaum L, Schechter T, Draiman B, Chitrit-Raveh E, et al. Expansion of the GRIA2 phenotypic representation: a novel de novo loss of function mutation in a case with childhood onset schizophrenia. J Hum Genet. 2021;66(3):339-43.

# Supplementary Figures and Tables:

A.

B.

C.

Supplementary Figure 1. Simplified representation of the Costa Rican TS pedigree, split into three images to show the complex relationships between the nineteen sequenced individuals (boxed in red) and the six founder pairs (A-F) (boxed in blue): A. Pedigree image of founder pairs A,B,C,D and E and their descendants, including the eleven sequenced individuals 1, 5, 6, 8, 9, 14, 15, 16, 17, 18 and 19; B. Pedigree image of founder pairs C, D and F and their descendants, including the sequenced individuals 1, 2, 3, 4, 6 and 20; C. Pedigree image of founder pairs A,B,C, and D and their descendants, including the nine sequenced individuals 6, 7, 8, 9, 10, 11, 12, 13 and 19.

Supplementary Table 1. Description of the twenty individuals selected for WGS, including their phenotype and which of the six founder pairs (A-F) they are descended from. The DNA for individual 14 failed quality control checks and was not taken forward for WGS. Phenotype abbreviations: TS (Tourette syndrome confirmed); OCD (obsessive compulsive disorder confirmed); OCD prob (probable OCD diagnosis); ADHD (attention deficit hyperactivity disorder confirmed); ADHD prob (probable ADHD diagnosis); CMVT (chronic motor/verbal tic disorder)

Supplementary Figure 2. PCA plots against the 1000 genomes Phase 3 dataset for: A. the unaffected founders from the Costa Rican BD study; B. the Costa Rican super controls; and C. the TS pedigree individuals. These plots show that both control cohorts, similar to the TS pedigree individuals, are ancestrally most similar to the 1000 Genomes Admixed American (AMR) population samples.


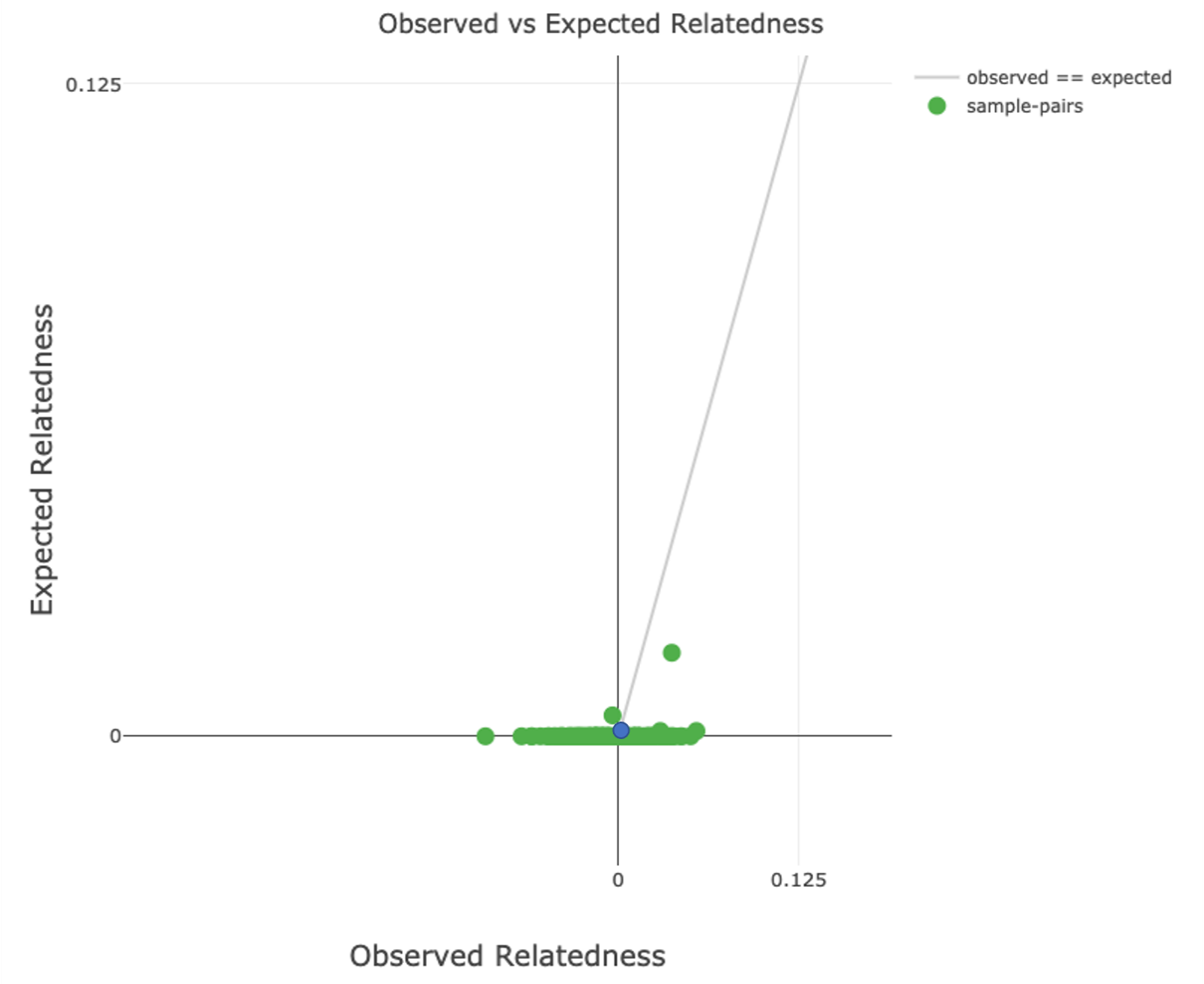


Supplementary Figure 3. Peddy Ped-check of TSL3 pedigree individuals (green circles) vs expected relatedness for 5^th^ cousins (blue circle).

Supplementary Table 2. Detailed description of all 19 IBD haplotypes that are > 1Mb in length, carried by at least three individuals sharing at least one common ancestor: the genomic location of the haplotypes; carrier IDs; all founder pairs related to each carrier; the main founders (most frequent founder pair(s) for each haplotype); the number of haplotype carriers descended from the main founder; and the frequency of each haplotype in two control cohorts (the Costa Rican bipolar disorder cohort unaffected founders and the Costa Rican Super Controls).

Supplementary Table 3. Summary of haplotype fine-mapping analysis showing the total number variants on each of the eleven IBD haplotypes and the proportion of phased vs not phased variants. Further subdivided into the set of variants where the alleles are unique to the haplotype (not present in any other pedigree individuals) and where the haplotype allele frequency is less than: 0.05 (low-frequency subset); 0.01 (rare subset); and 0.001 (ultra-rare subset). Within each of the low-frequency, rare and ultra-rare sets any variants representing an amino acid substitution are counted in brackets.

Supplementary Table 4. All putative deleterious variants that survived the variant filtering pipeline, annotated with functional annotations (VEP Consequence; VEP Impact; associated gene symbol; amino acid substitutions; as well as SIFT, PolyPhen, CADD, pLOF and ncER scores) and GnomAD minor allele frequencies across all population sets: AMR (Latino/Admixed American); MAX (maximum allele frequency across any population); ASJ (Ashkenazi Jewish); EAS (East Asian); FIN (Finnish); NFE (non-Finnish European); SAS (South Asian) and OTH (Other). All five variants are rare (MAF < 0.01) or ultra-rare (MAF < 0.001) in all populations.

Supplementary Figure 4. Output from the STRING network analysis of the brain-expressed genes harboring rare and ultra-rare haplotype-specific variants, showing all protein interaction networks (both physical and functional interactions). The top four protein-coding genes from the variant filtering analysis (*RAPGEF1*, *NASP*, *ERBB4* and *IKZF2*) are circled in red. Lines of different colors connecting the proteins into networks reflect the different types of evidence supporting their interaction: known interactions; predicted interactions; text-mining; co-expression data; and protein homology.

Supplementary Figure 5. The haplotype-specific STRING networks containing the top protein-coding genes from the variant filtering analysis: *RAPGEF1*, *ERBB4,* *IKZF2* and *NASP*. *RAPGEF1* and *ERBB4*, along with ten other brain-expressed genes harboring rare and ultra-rare haplotype-specific variants form a single Network with an enrichment of 102 GO terms with the most significant term being *regulation of migration*. *IKZF2* is part of a network of five genes, while *NASP* is part of a two gene network with *SMC2*, neither network having any significant GO terms.

Supplementary Table 5. Results of the PANTHER GO enrichment analysis for the network of 12 protein-coding genes containing *ERBB4* and *RAPGEF1*, showing the top ten enriched GO terms. Full list in Supplementary Data.

Supplementary Table 6. Results of the PANTHER GO enrichment analysis for all 66 brain-expressed protein-coding genes containing rare and ultra-rare haplotype-specific variants. Showing the top 50 of the 467 GO terms with un-corrected p-values < 0.05. See Suppl Data for full results.

Supplementary Table 7. Summary of the investigation of the haplotype genes based on the PsychENCODE cross-disorder gene expression analysis, showing: genes that have been shown to be up or down regulated in disorder cases (ASD, BD or SCZ) versus controls; genes with disorder-specific isoforms; whether the gene is part of a gene co-expression or isoform co-expression module; FDR corrected association of the module with ASD, BD and SCZ; whether the gene is in the top 50 genes for the module, module-specific cell-type enrichment, module specific enrichment in GWAS hits; the number of GO terms associated with the module; the top GO term for the module; and any other notable terms from the top 10 GO associated with the module.
